# An isolated elevation in blood urea level is not ‘uraemia’ and not an indication for renal replacement therapy in the ICU

**DOI:** 10.1186/s13054-017-1868-x

**Published:** 2017-11-13

**Authors:** Jack Mackenzie, Bobby Chacko

**Affiliations:** 1 School of Medicine and Public Health, University of Newcastle, Newcastle, NSW Australia; 2 Nephrology and Transplantation Unit, John Hunter Hospital, Newcastle, NSW 2310 Australia

**Keywords:** Uraemia, RRT, ICU

## Abstract

The decision to initiate renal replacement therapy (RRT) and the optimal timing for commencement is a difficult decision faced by clinicians when treating acute kidney injury (AKI) in the intensive care setting. Without clinically significant ureamic symptoms or emergent indications (electrolyte abnormalities, volume overload) the timing of RRT initiation remains contentious and inconsistent across health providers. Current trends of initiating RRT in the ICU are often based on isolated blood urea levels without clear guidelines demonstrating an upper limit for treatment. Although the appropriate upper limit remains unclear, it is reasonable to conclude that a blood urea level less than 40 mmol/L is not in itself an indication for RRT, especially in the absence of supporting evidence of kidney impairment (anuria, elevated serum creatinine), presenting a welcome reminder to treat the patient and not a number.

## Introduction

The decision to initiate renal replacement therapy (RRT) and the optimal timing for commencement is a difficult decision clinicians face when treating acute kidney injury (AKI) in the intensive care setting. Without clinically significant ureamic symptoms or emergent indications (electrolyte abnormalities, volume overload) the timing of RRT initiation remains contentious and inconsistent across health providers. The issue is further complicated by several randomised control trials (RCTs) that investigated early versus delayed prescription of RRT producing conflicting results [1–5].

Despite the common assumption that elevated urea requires urgent dialysis, treating AKI on serum urea alone is difficult due to variations in the base urea level in the presence of metabolic instability and variations in urea generation [6]. The clinical signs of pathological uraemia (pericarditis, pleuritis, encephalopathy and bleeding) are well established as indications to start haemodialysis [6], but therapy often commences prior to their development.

It is also common practice to delay haemodialysis for an indefinite period in the absence of these specific uraemic complications due to the risks associated with RRT [6]. However, the question remains, when is the most appropriate time to initiate therapy based on serum urea levels without other apparent indications?

## Discussion

Despite early meta-analysis suggesting early RRT improves survival in critical illness [7], more recent analysis does not support that view [8, 9]. The most comprehensive data regarding optimal timing for RRT initiation comes from a 2017 meta-analysis of RCTs assessing early versus late initiation of RRT in patients with AKI [8] that concluded there was no added benefit of early initiation with respect to 30, 60, and 90-day mortality, overall ICU and hospital mortality, and dialysis dependence. Older studies have demonstrated increased mortality when serum urea reaches higher ranges (50.7–71.4 mmol/L) [10], which perhaps established the tendency to initiate RRT therapy at lower levels.

Evaluation of the available data led to the proposal of serum urea > 35.7 mmol/L as an absolute indication for RRT [11] but no recent RCTs have looked at urea as an independent variable for initiation. The only study found to consider serum urea in isolation confirmed a declining trend of threshold urea level for RRT initiation and no association with in-hospital mortality [12]. Most recent studies did, however, implement an upper limit at which interventional RRT was initiated Table 1.

**Table 1:** Collation of recent studies with blood urea level criteria in exclusion or intervention

Study	Year	Urea exclusion criteria	Late RRT urea intervention threshold
Zarbock et al. [5]	2016	None	Serum urea > 100 mg/dL (35.7 mmol/L)
Gaudry et al. [3]	2016	Urea blood > 112 mg/dL (40 mmol/L)	Urea blood > 112 mg/dL (40 mmol/L)
Combes et al. [2]	2015	None	Serum urea > 36 mmol/L
Jamale et al. [4]	2013	Life-threatening uremic complications (alteration of higher mental function attributable to uraemia, and pericarditis)	Uremic nausea
Bagshaw et al. [14]	2009	None	Serum urea > 24.2 mmol/L
Bouman et al. [1]	2002	None	Serum urea > 40 mmol/L
Pursnani et al. [15]	1997	Urea blood > 120 mg/dL (42.8 mmol/L)	Serum urea > 40 mmol/L

As no recent RCTs assess urea independently, it is reasonable to extrapolate the correlating urea levels to make a plausible appraisal of what level is acceptable to commence RRT. The recently examined upper limit was > 35.7 mmol/L to the onset of clinical signs or symptoms, although most studies initiated treatment no higher than 40 mmol/L. With evidence that no added benefit is derived from early therapy, in the absence of emergent indications a serum urea level of 40 mmol/L is therefore not unreasonable.

The practical implications are balancing the theoretical assumption that early initiation of RRT may lead to earlier removal of uraemic toxins, against the possibility that delayed treatment may result in spontaneous recovery and avoidance of RRT entirely. The KDIGO guidelines conclude that it is unclear whether the risks outweigh the benefits and that RRT should be initiated emergently when life threatening changes in fluid, electrolyte and acid–base balance exist [7]. Furthermore, it recommends considering the broader clinical context, the presence of modifiable conditions and trends of laboratory tests rather than serum urea and creatinine thresholds alone [7].

Without definitive guidelines, there is sufficient evidence to suggest that initiation of RRT in a patient with a urea level less than 40 mmol/L in an otherwise stable clinical context is erroneously exposing them to risks of dialysis without any increased survival benefit. Furthermore, a reasonable argument can be made that even above 40 mmol/L, delaying RRT may be warranted in an otherwise clinically well patient with preserved kidney function.

Correlating clinical symptoms with blood urea levels in the ICU to aid the decision to commence RRT is complicated by many factors that have been identified as contributors to elevated urea levels such as dehydration, increased tubular urea reabsorption, heart failure, glucocorticoid use, gastrointestinal haemorrhage and exogenous protein sources (dietary intake, PEG feeds and colloids). At moderate serum urea levels (21.4 mmol/L) these factors do not appear to elicit ureamic symptoms in isolation from severely impaired kidney function [13].

Despite reliance on historical data and lack of recent quality evidence, it is plausible that, in the context of established kidney injury, the onset of ureamic symptoms is likely when the blood urea level is greater than 50 mmol/L. However, given the poor correlation with clinical symptoms, the multifactorial nature of AKI and variation in production and excretion, serum urea alone remains a poor indicator of ureamic toxicity, especially in the presence of additional urea-elevating factors, or the absence of other symptomatic or biochemical markers of severe kidney impairment.

## Conclusions

Currently there is no well-established upper-limit of serum urea that serves as an indication for initiation of RRT in AKI. Using urea in isolation is difficult and potentially flawed given the variation in base level and production rates amongst different populations as well as it’s negligibility as an accurate measure of metabolite toxicity. Furthermore, AKI is a multifactorial condition and it is likely to present earlier with other concerning metabolic or fluid overload abnormalities requiring dialysis rather than isolated elevated blood urea levels. Studies have considered the appropriate timing of initial RRT including early and delayed/late commencement of therapy, with a recent meta-analysis concluding there is no added benefit. Most studies initiated RRT once blood urea level exceeded 40 mmol/L, which is reflected in clinical practice despite evidence to suggest it does not correlate well with onset of uraemic symptoms.

Although the appropriate upper limit remains unclear, it is reasonable to conclude that a blood urea level less than 40 mmol/L is not in itself an indication for RRT therapy, especially in the absence of supporting evidence of kidney impairment (anuria, elevated serum creatinine), presenting a welcome reminder to treat the patient and not a number.

## Abbreviations

AKI: Acute kidney injury
CI: Confidence interval
CKD: Chronic kidney disease
ICU: Intensive care unit
KDIGO: Kidney Disease: Improving Global Outcomes
RCT: Randomised control trial
RR: Relative risk
RRT: Renal replacement therapy

## References

[1] Bouman CS, Oudemans-Van Straaten HM, Tijssen JG (2002). Effects of early high-volume continuous venovenous hemofiltration on survival and recovery of renal function in intensive care patients with acute renal failure: a prospective, randomized trial. Crit Care Med.

[2] Combes A, Bréchot N, Amour J (2015). Early high-volume hemofiltration versus standard care for post-cardiac surgery shock. The HEROICS Study. Am J Respir Crit Care Med.

[3] Gaudry S, Hajage D, Schortgen F (2016). Initiation strategies for renal-replacement therapy in the intensive care unit. N Engl J Med.

[4] Jamale TE, Hase NK, Kulkarni M (2013). Earlier-start versus usual-start dialysis in patients with community-acquired acute kidney injury: a randomized controlled trial. Am J Kidney Dis.

[5] Zarbock A, Kellum JA, Schmidt C (2016). Effect of early vs delayed initiation of renal replacement therapy on mortality in critically ill patients with acute kidney injury: the ELAIN Randomized Clinical Trial. JAMA.

[6] Kidney Disease: Improving Global Outcomes (KDIGO) Acute Kidney Injury Work Group (2012). KDIGO clinical practice guideline for acute kidney injury. Kidney Int Suppl.

[7] Karvellas CJ, Farhat MR, Sajjad I, Mogensen SS, Leung AA, Wald R (2011). A comparison of early versus late initiation of renal replacement therapy in critically ill patients with acute kidney injury: a systemic review and meta-analysis. Crit Care.

[8] Bhatt GC, Das RR (2017). Early versus late initiation of renal replacement therapy in patients with acute kidney injury-a systematic review & meta-analysis of randomized controlled trials. BMC Nephrol.

[9] Lai TS, Shiao CC, Wang JJ (2017). Earlier versus later initiation of renal replacement therapy among critically ill patients with acute kidney injury: a systematic review and meta-analysis of randomized controlled trials. Ann Intensive Care.

[10] Nascimento GV, Gabriel DP, Abrao JM, Balbi AL (2010). When is dialysis indicated in acute kidney injury?. Ren Fail.

[11] Gibney N, Hoste E, Burdmann EA (2008). Timing of initiation and discontinuation of renal replacement therapy in AKI: unanswered key questions. Clin J Am Soc Nephrol.

[12] De Corte W, Vanholder R, Dhondt AW (2011). Serum urea concentration is probably not related to outcome in ICU patients with AKI and renal replacement therapy. Nephrol Dial Transplant.

[13] Meyer TW, Hostetter TH, Skorecki K, Chentow GM (2016). The pathophysiology of uremia. Brenner and Rector’s the kidney.

[14] Bagshaw SM, Uchino S, Bellomo R (2009). Timing of renal replacement therapy and clinical outcomes in critically ill patients with severe acute kidney injury. J Crit Care.

[15] Pursnani ML, Hazra DK, Singh B, Pandey DN (1997). Early haemodialysis in acute tubular necrosis. J Assoc Physicians India.

